# B Cell Activating Factor of the Tumor Necrosis Factor Family (BAFF) Behaves as an Acute Phase Reactant in Acute Pancreatitis

**DOI:** 10.1371/journal.pone.0054297

**Published:** 2013-01-14

**Authors:** Georg Pongratz, Hannah Hochrinner, Rainer H. Straub, Stefanie Lang, Tanja Brünnler

**Affiliations:** 1 Department of Internal Medicine I, University Hospital Regensburg, Regensburg, Germany; 2 Emergency Centre, Hospital of the Order of St.John of God Regensburg, Regensburg, Germany; Centro di Riferimento Oncologico, IRCCS National Cancer Institute, Italy

## Abstract

**Objective:**

To determine if B cell activating factor of the tumor necrosis factor family (BAFF) acts as an acute phase reactant and predicts severity of acute pancreatitis.

**Methods:**

40 patients with acute pancreatitis were included in this single center cohort pilot study. Whole blood and serum was analyzed on day of admission and nine consecutive days for BAFF, c-reactive protein (CRP), interleukin-6 (IL-6), procalcitonin (PCT), and leucocyte numbers. Different severity Scores (Ranson, APACHE II, SAPS II, SAPS III) and the clinical course of the patient (treatment, duration of stay, duration ICU) were recorded.

**Results:**

Serum BAFF correlates with CRP, an established marker of severity in acute pancreatitis at day of admission with a timecourse profil similar to IL-6 over the first nine days. Serum BAFF increases with Ranson score (Kruskal-Wallis: Chi2 = 10.8; p = 0.03) similar to CRP (Kruskal-Wallis: Chi2 = 9.4; p = 0.05 ). Serum BAFF, IL-6, and CRP levels are elevated in patients that need intensive care for more than seven days and in patients with complicated necrotizing pancreatitis. Discriminant analysis and receiver operator characteristics show that CRP (wilks-lambda = 0.549; ROC: AUC 0.948) and BAFF (wilks-lambda = 0.907; ROC: AUC 0.843) serum levels at day of admission best predict severe necrotizing pancreatitis or death, outperforming IL-6, PCT, and number of leucocytes.

**Conclusion:**

This study establishes for the first time BAFF as an acute phase reactant with predictive value for the course of acute pancreatitis. BAFF outperforms established markers in acute pancreatitis, like IL-6 and PCT underscoring the important role of BAFF in the acute inflammatory response.

## Introduction

B cell activating factor of the tumor necrosis factor family (BAFF) is a member of the TNF superfamily (alternative names are B lymphocyte stimulator (BLyS), TALL-1, zTNF4, THANK and TNFSF13B). Increased systemic levels of BAFF in serum and other body fluids like bronchoalveolar lavage, synovial fluid and gut lavage have been associated with disease activity of many autoimmune (e.g. systemic lupus erythematosus, rheumatoid arthritis, Sjögren’s syndrom, psoriatic arthritis [1], systemic sclerosis, myasthenia gravis, celiac disease, autoimmune hepatitis, primary biliary cirrhosis, bullous pemphigoid), allergic diseases (asthma, allergic rhinitis), and malignant diseases like B-CLL and multiple myeloma (reviewed in [2]). Also, some infections like HIV, EBV and Hepatitis C seem to go along with increased BAFF serum levels [2].

BAFF can act on target cells via three different receptors, namely BAFF-Receptor (BAFF-R), transmembrane activator and calcium modulator and cyclophilin interactor (TACI) and B cell maturation protein (BCMA, reviewed in [3]). These receptors are expressed on B cells to various degrees depending on the B cell maturation stage. However, also activated T cells and some non-lymphoid cell types e.g. synovial fibroblasts express receptors for BAFF [4], [5]. A proliferation induced ligand (ARPIL) also promotes B cell survival and shares binding to TACI and BCMA receptors with BAFF [3]. The physiological impact of BAFF on the B cell compartement is manifold [3]. At early B cell stages, stimulation of BAFF-R on transitional B cells generates a survival signal, leading to a less stringent selection process against autoantigens. This function of BAFF seems to play an important role in the brake of B cell tolerance during the development of some autoimmune diseases e.g. SLE [6]. Supporting this notion, clinical trials using BAFF antagonists, e.g. Belimumab show effectiveness in the treatment of SLE (reviewed in [7]). At mature B cell stages the presence of BAFF increases class-switch recombination to IgG, IgE and IgA [3], which might explain the association of high serum BAFF levels with allergic diseases. On the other hand, BAFF acts on the T cell compartment and favors Th1 and Th17 responses while inhibiting Th2 responses [8].

Taken together BAFF influences the inflammatory response in many places and seems to be upregulated during inflammatory processes regardless of the cause (allergic diseases, infectious diseases, autoimmunity, malignancy). This led us to the hypothesis that BAFF is an acute phase protein similar to CRP.

To test this hypothesis we measured several parameters of inflammation and BAFF serum levels in a prospective study of patients with acute pancreatitis in the early stages of disease. We have chosen this approach because pancreatitis in its early stages can be regarded as a model for the pathophysiological process of severe inflammation without confounding factors like infectious agents, allergies, malignancies or autoimmunity. Additionally, this approach gave us the opportunity to evaluate BAFF as compared to CRP, IL-6, PCT, and number of leucocytes as a predictor of severity and course of acute pancreatitis.

## Methods

### Ethics Statement

The ethics committee of the University Regensburg approved the study (Nr. 08/008). The study was registered with ClinicalTrials.gov Identifier: NCT00699933. Patients were included in the study after obtaining written informed consent from the patient or guardian.

### Study Population

Over a period of 30 months, 50 patients with acute pancreatitis were included in this prospective, single-centre cohort study. Diagnosis of acute pancreatitis was based on clinical assessment (typical upper abdominal pain associated with nausea and/or vomiting) and an elevated level (>3 x ULN) of serum lipase. All patients with symptoms for more than 48 h or diagnosis of autoimmune diseases were excluded, resulting in 40 patients who were included in the final analysis. For three patients, BAFF levels at day 0 were not available, therefore analyses using BAFF levels at day 0 only included 37 patients. Patient characteristics are summarized in table 1. Most patients were diagnosed with alcohol induced (n = 17) or biliary pancreatitis (n = 12). Other causes included post-ERCP pancreatitis (n = 4), drug-induced pancreatitis (n = 3), triglycerid-induced pancreatitis (n = 2), pancreatitis due to pancreas divisum (n = 1), and idiopathic pancreatitis (n = 1).

**Table 1:** Patient characteristics.

	edematous	necrotizing	total
**female (N)**	7	5	12
**male (N)**	15	13	28
**all (%)**	55	45	100
**age (yrs., SD)**	55.7 (18.8)	59.5 (17.4)	55.4 (17.9)
**BMI (kg/m∧2, SD)**	29.9 (7.3)	27.3 (5.4)	29.4 (6.5)
**death (N, %)**	0 (0)	3 (16.7)	3 (7.5)
**duration (days, SD)**	12.1 (9.5)	40.0 (37.6)	25.0 (29.6)
**ICU >7 days (N)**	1	8	9
**etiology (N, %)**			
** biliary**	13	4	17 (42.5)
** alcohol**	4	8	12 (30.0)
** other**	5	6	11 (27.5)
**treatment (N, %)**			
** antibiotics**	11	14	25 (62.5)
** drain**	0	7	7 (17.5)
** necrosectomy**	0	2	2 (5)
**scores (Avg, SD)**			
** Ranson**	2.1 (1.5)	3.0 (2.0)	2.5 (1.7)
** IMRIE**	1.0 (1.0)	2.2 (1.5)	1.6 (1.4)
** SAPS II**	14.5 (10.4)	25.5 (17.0)	19.5 (14.7)
** SAPS III**	14.0 (10.8)	24.9 (17.0)	18.9 (14.8)
** APACHE II**	5.9 (4.1)	10.9 (7.3)	8.2 (6.2)

Sonography of the abdomen, computed tomography, and magnetic resonance imaging (MRI) were performed within the first 72 hours to establish a diagnosis of necrotizing vs. edematous pancreatitis.

### Laboratory Testing

On admission and for the following 9 days venous blood samples were drawn on a daily basis. Full blood samples were centrifuged at 5000 U/min for 10 minutes and serum aliquots were immediately stored at –80°C until analysis. BAFF and APRIL was assayed by commercially available antigen-capture enzyme-linked immunosorbent assay (ELISA; R&D Systems, Minneapolis, USA).

C-reactive protein (CRP, determined by ADVIA 1800, Siemens), serum Interleukin-6 (IL-6, determined by Immulite 2000 XPi, Siemens), procalcitonin (PCT, determined by Centaur, Siemens), and the total number of leucocytes (Sysmex XE 5000, Sysmex ) were determined immediately after the blood draw by standardized routinely used procedures as indicated.

### Statistical Analysis

Sigma Plot (Systat Software, V 11.0) was used to determine correlations using spearman rank test. Differences between multiple groups were determined by nonparametric- (Kruskal wallis) or parametric (general linear model) analysis of variance using PASW Statistics (Version 18.0, Polar Engeneering) depending on the distribution of the data. Post hoc tests (Mann-Whitney U Test corrected for number of tests, or Holm-Sidak Test) were used for multiple comparisons in cases of overall differences detected by ANOVA (significance level p = 0.05). PASW Statistics 18 was also used for generating and analyzing a discriminant model and analyzing receiver-operator characteristics (ROC) of several tests for prediction of severity in acute pancreatitis. Displayed error bars represent the standard error of the mean. P-values below or equal to 0.05 were regarded as significant.

## Results

### Serum BAFF but not APRIL Correlates with CRP in Patients with Acute Pancreatitis at the Day of Admission

**Figure 1:**
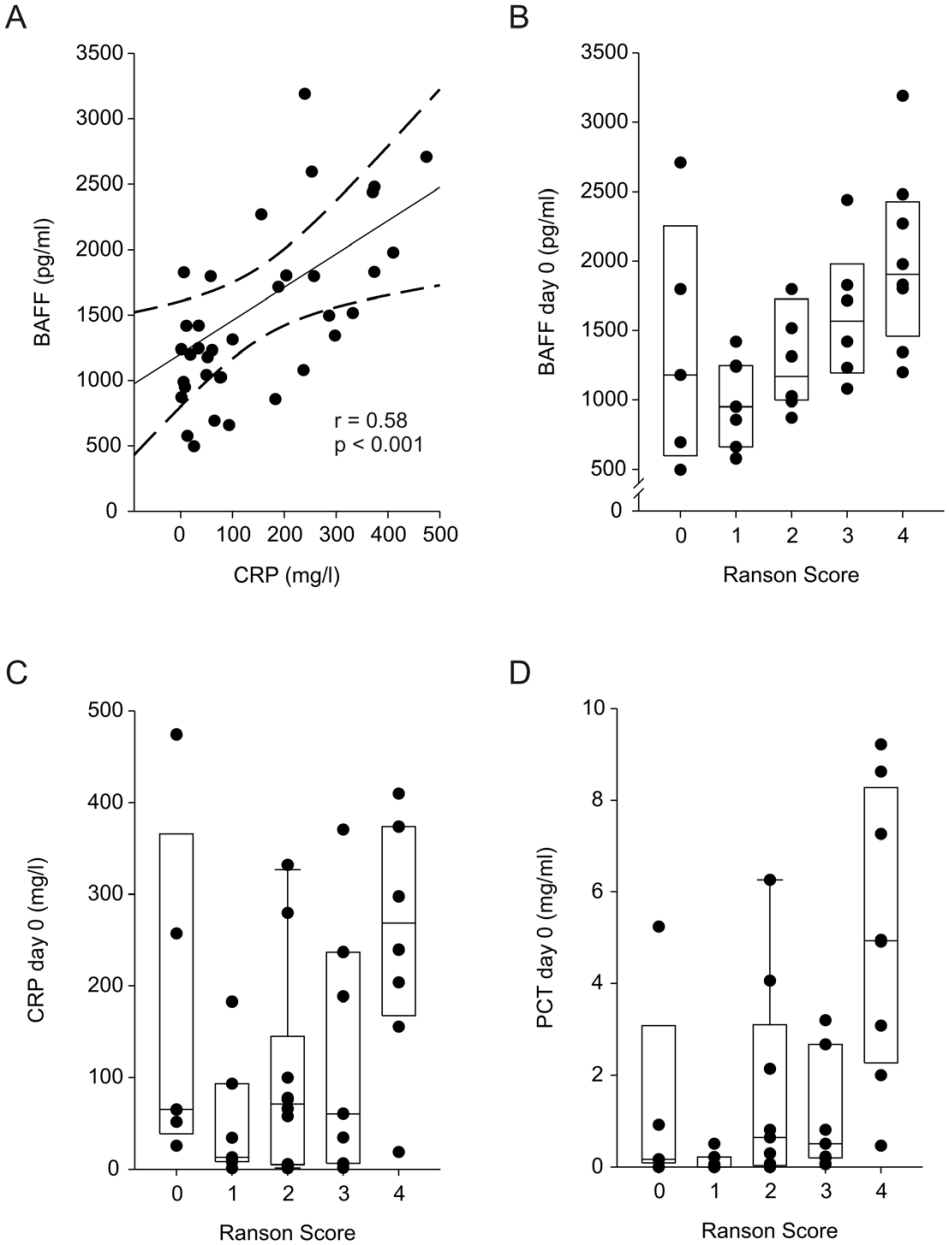
Relationship of BAFF, CRP, and PCT serum levels, and Ranson score. (A) Scatter plot for serum BAFF (pg/ml) and CRP (mg/l). Correlation coefficient was determined by Spearman procedure. Linear regression line (solid line), 95% confidence interval (dashed lines). (B) Different median values of serum BAFF (pg/ml, solid lines) at different categories of Ranson Score. Each dot represents value of one patient at day of admission. An overall significant difference between the groups was determined by Kruskal-Wallis statistic (Chi^2^ = 10.8; p = 0.03). (C) Different median values of CRP (solid lines) at different categories of Ranson Score. Each dot represents value of one patient at day of admission. An overall significant difference between the groups was determined by Kruskal-Wallis statistic (Chi^2^ = 9.4; p = 0.05). (D) Different median values of PCT (solid lines) at different categories of Ranson Score. Each dot represents value of one patient at day of admission. An overall significant difference between the groups was determined by Kruskal-Wallis statistic (Chi^2^ = 15.5; p = 0.004).

As shown in Figure 1A, CRP Serum levels at the timepoint of admission (day 0) correlated with serum BAFF levels (r = 0.58, p<0.001). At day 0 there was no correlation between serum BAFF levels and number of leucocytes (r = 0.07, p = 0.6) or serum IL-6 (r = 0.19, 0.25). However, serum BAFF and IL-6 showed a significant correlation when analyzed at the first day after admission (r = 0.41, p = 0.009).

ARPIL also promotes B cell survival and shares binding to TACI and BCMA receptors with BAFF [3]. Therefore, we also determined APRIL serum levels in our patients. In contrast to BAFF, APRIL measured at day 0 does not show any correlation with CRP (r = 0.32, p = 0.1), IL-6 (r = 0.27, p = 0.16) or leucocytes (r = 0.30, p = 0.12).

These results indicate that serum BAFF but not APRIL levels are an early marker of inflammation, like serum CRP.

### Serum BAFF and CRP Increases with Increasing Ranson Score

Ranson score is an established predictor of severity of acute pancreatitis [9]. We therefore determined if the median values of BAFF and CRP, respectively, differ between the patients with different Ranson Scores. As depicted in Figure 1B, serum BAFF levels significantly increase (Kruskal-Wallis: Chi^2^ = 10.8; p = 0.03) with increasing Ranson score from 0 to 4. Ranson score 5, 6, and 8 were only reached by one patient each and were excluded from analysis. No patient reached a Ranson score greater than 8. CRP serum levels (Kruskal-Wallis: Chi^2^ = 9.4; p = 0.05; Figure 1C) and PCT serum levels (Kruskal-Wallis: Chi^2^ = 15.5; p = 0.004; Figure 1D) also show an increase with Ranson score. In contrast IL6 serum levels (Kruskal-Wallis: p = 0.18) and leucocyte counts (Kruskal-Wallis: p = 0.19) did not show significant differences in their distribution among the different Ranson score categories (data not shown). Therefore, out of the determined inflammatory markers at the day of admission, BAFF, PCT and CRP serum levels might have a predictive value for the course of acute pancreatitis.

### Serum BAFF, PCT, IL-6, and CRP Levels are Higher in Patients that need Intensive Care for more than Seven Days

**Figure 2:**
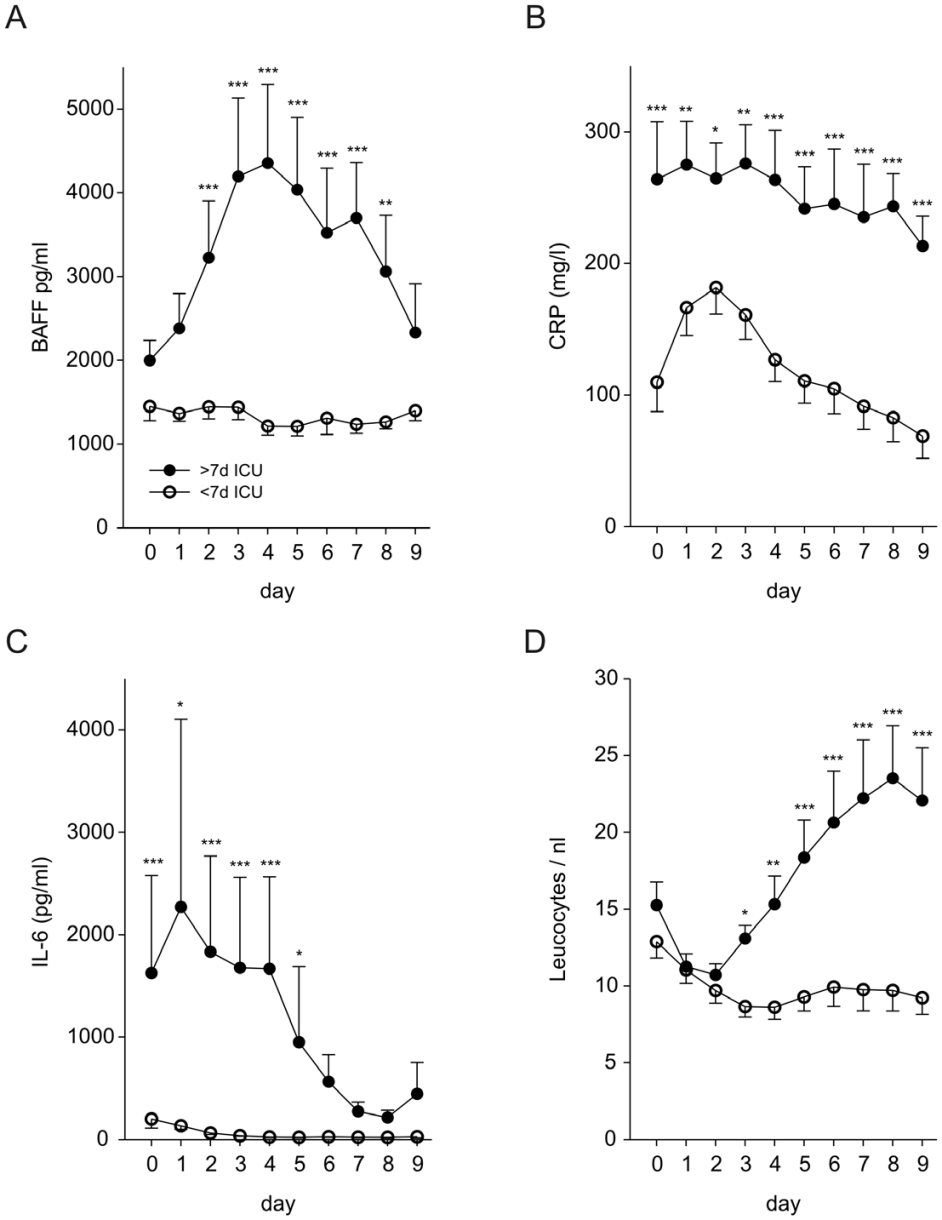
Time course of serum BAFF, CRP, IL-6, and leucocyte counts after stratification of patients according to the duration of intensive care. Serum levels of BAFF (pg/ml, A), CRP (mg/l, B), IL-6 (pg/ml, C), and leucocyte counts (/nl, D) after stratification of patients into cases needing more than one week or less or equal to one week of intensive care. Differences between groups (BAFF: p<0.001; CRP: p<0.001; IL-6: p = 0.001; Leucocyte count: p<0.001) were determined by general linear modeling and Student-Newman-Keuls method (* p<0.05, ** p<0.01, *** p<0.001). Error bars respresent the S.E.M.

To further invastigate the predictive value of the determined inflammatory parameters, patients were stratified in one group that needed intensive care for more than seven days and one group that needed seven days or less, respectively. This stratification approach was chosen because one of the best characterized tools to predict severity of acute pancreatitis, the Ranson score, was developed using the same stratification procedure to distinct between severe and less severe courses of acute pancreatitis [10], [11]. Serum levels of BAFF, CRP, IL-6 and leucocyte numbers were plotted over the first nine days after admission to the hospital (Figure 2). PCT values mimicked the course of IL-6, however, showed a greater variablility (data not shown). BAFF, CRP, IL-6 and PCT serum levels are higher in patients that needed intensive care for more than one week (Figure 2A–C, data not shown). Leukocyte counts also distinct between the two groups of patients, however, a significant difference was not reached before day four after admission (Figure 2D). Notably, there is a significant increase in the level of serum BAFF in the group of patients that will need more than seven days intensive care, whereas, patients needing less than seven days intensive care show constant BAFF levels over time (Figure 2A). In contrast, patients needing less than seven days of intensive care show an increase in CRP levels over the first two days after admission and a decrease thereafter, whereas CRP levels in severe cases stay constantly high (Figure 2B). Serum IL-6 levels are higher from the beginning in patients that will need more than seven days of intensive care and drop from day 4 after admission (Figure 2C).

### Serum BAFF, IL-6 and CRP Levels are Increased in Patients Who Show a Severe Course of Acute Necrotizing Pancreatitis or Die

**Figure 3:**
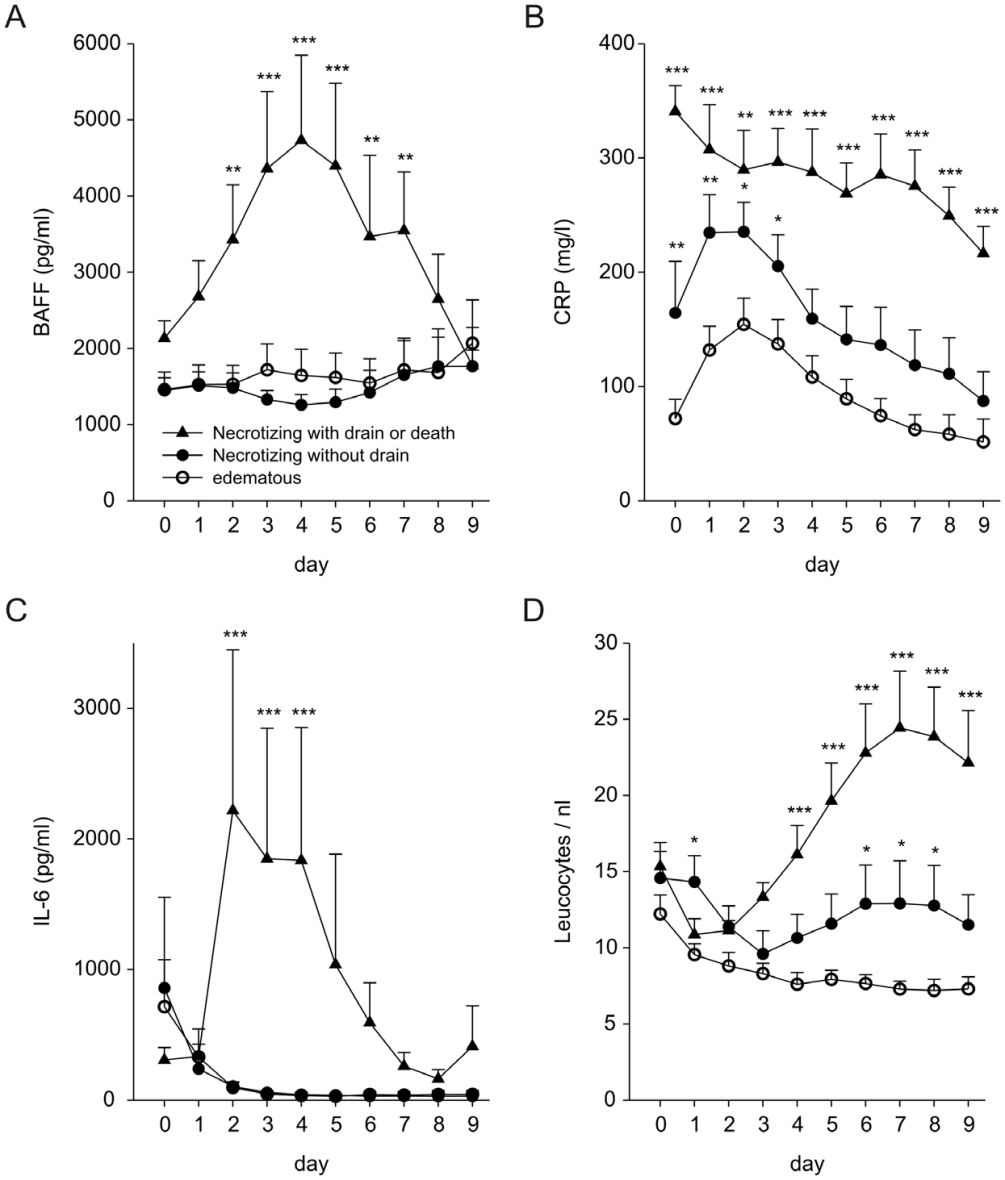
Time course of serum BAFF, CRP, IL-6, and leucocyte counts after stratification of patients in edematous, necrotizing, and severe necrotizing pancreatitis or death. Serum levels of BAFF (pg/ml, A), CRP (mg/l, B), IL-6 (pg/ml, C), and leucocyte counts (/nl, D) after stratification of patients into cases with edematous pancreatitis (EP, open circles), necrotizing pancreatitis (NP, solid circles), and severe necrotizing pancreatitis or death (sNPoD, solid triangles). Differences between groups (BAFF: p<0.001; CRP: p<0.001; IL-6: p<0.001; Leucocyte count: p<0.001) were determined by general linear modeling and Student-Newman-Keuls method. P-values represent differences as compared to EP (* p<0.05, ** p<0.01, *** p<0.001). Error bars respresent the S.E.M.

In a second approach we stratified our patients according to the severity and course of disease. To achieve this, we grouped patients with edematous pancreatitis (EP), necrotizing pancreatitis without the need for drain or operation (NP), and necrotizing pancreatitis with the need for drain or death (sNPoD). The number of deaths was too low to generate an own variable, because only 3 out of 40 patients (7.5%) died during the observation period. BAFF, CRP, IL-6, and leucocyte counts are increased in patients with sNPoD as compared to patients with EP (Figure 3). PCT serum levels showed a high degree of variability, especially in patients with sNPoD, however we could not detect a significant difference between the groups (general linear model, p = 0.25). There are no significant differences between serum levels of IL-6 and BAFF, respectively in patients with EP or NP over the observed time period (Figure 3A, C). However, BAFF levels are increased in patients with sNPoD as compared to NP or EP from day 2 after admission until day 7 (Figure 3A). Serum IL-6 levels are also increased in patients with sNPoD as compared to NP or EP from day 2 until day 4 (Figure 3C). CRP serum levels show a decline over time and differ between all groups with a significant difference starting from the day of admission (Figure 3B). Leucocyte counts also distinct all three groups, however, only from day 6 to 8 after admission (Figure 3D). Remarkably, a rise of leucocytes seems to be a better marker of complicated necrotizing pancreatitis than a change in CRP levels.

Taken together, BAFF as well as IL-6 best distinct between severe cases of acute pancreatitis (sNPoD) and less severe courses of disease (NP or EP), regardless if necrotizing or not. On the other hand, CRP might have an additional value in the distinction between EP and NP. In contrast, PCT serum levels did not distinct between sNPoD and NP or EP, respectively, mainly due to a high degree of variability. Leucocyte counts also distinct between the groups, however, to late in the course of disease to help with therapeutic decisions. Another notable aspect is the increase of CRP serum levels during the first two days after admission in patients with EP or uncomplicated NP, which might mislead therapeutic decisions in these patients.

### CRP and Serum BAFF Measured at Day of Admission are the Best Predictors of a Severe Course of Pancreatitis

**Figure 4:**
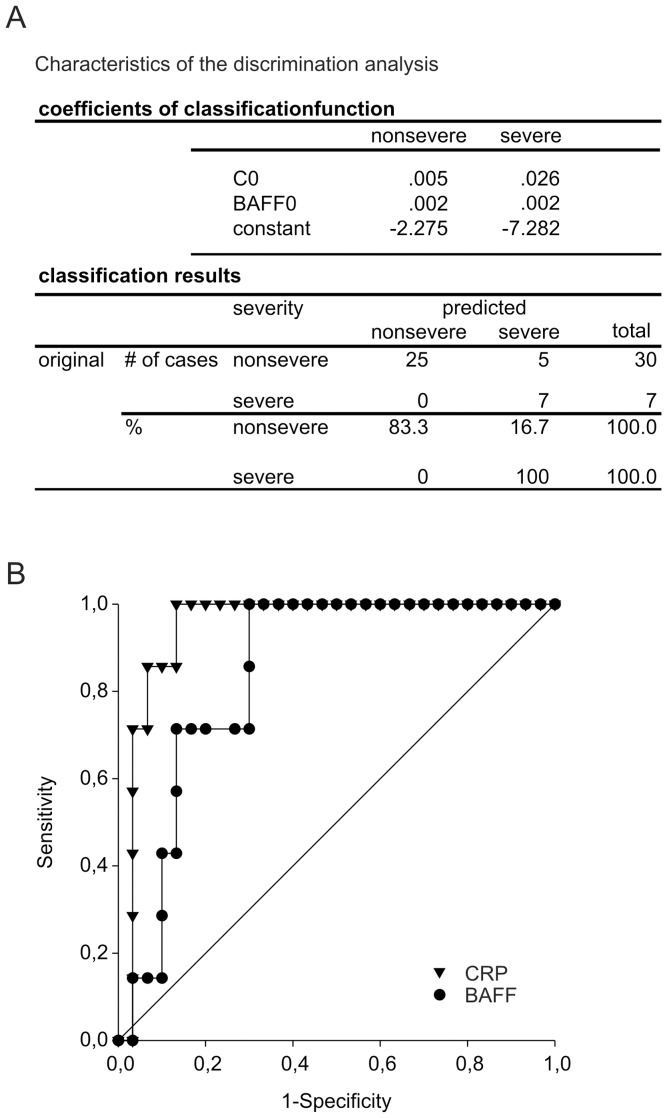
Discriminant analysis and receiver operator characteristic: Classification of severe (sNPoD) or nonsevere (NP or EP) cases using BAFF or CRP serum levels at day of admission, respectively. (A) Characteristic of the discrimination analysis. (B) Receiver operator characteristic (ROC) analysis for classification of patients developing severe pancreatitis (sNPoD) using serum BAFF (closed circles, AUC 0.843, cut-off: 1506 pg/ml, sensitivity 85.7%, specificity 70.0%) or CRP (triangles, AUC 0.948, cut-off: 271.6 mg/l, sensitivity 85.7%, specificity 93.3%). The solid line represents test-characteristic for a nonspecific test.

Discriminant analysis was performed to further investigate the value of serum BAFF, IL-6, CRP, PCT, and leukocyte counts measured at the day of admission to predict a severe course of acute pancreatitis (sNPoD). Test of equality of group means indicated that neither PCT (p = 0.84; wilks-lambda = 0.999) nor IL-6 (p = 0.43; wilks-lambda = 0.982) serum levels nor leucocyte counts (p = 0.21; wilks-lambda = 0.955) at day of admission would contribute to a discriminant model. Whereas, BAFF serum levels (p = 0.06; wilks-lambda = 0.907) showed a statistical trend and CRP (p<0.001; wilks-lambda = 0.549) a significant difference at admission between patients with severe course (sNPoD) as compared to patients developing less severe disease (NP or EP). Therefore, serum CRP, and BAFF at day of admission were included to generate a model that would discriminate between severe and less severe courses of acute pancreatitis. Using these variables we were able to generate a model with good discriminatory capacity between severe and non-severe cases (canonical correlation = 0.673., wilks-lambda = 0.55, Chi-Square = 20.47, p<0.001). Using cross validation, the model was able to classify 7 out of 7 patients (positive predictive value = 100%) correctly to the group of patients that will develop severe pancreatitis (Figure 4A). On the other hand the model only misclassified 16.7% of patients with less severe disease to the group of severe cases (false positive, Figure 4A). In a discriminant model only including CRP serum levels, the positive predictive value remained constant (100%), however, the false positve rate slightly increases (18.2%, data not shown). Taken together, these data show that CRP serum levels at the day of admission best predict the course of acute pancreatitis. Among serum IL-6, PCT, leucocyte counts, and serum BAFF measured at day of admission, only the addition of serum BAFF further improves the predictive capacity of the discriminant model.

To further analyze the classification properties of initial serum levels of BAFF, CRP, IL-6, PCT, and leucocyte counts we determined the receiver-operator characteristic of each variable with respect to classification of cases that will show a severe course of disease (Figure 4B). As expected from the discriminant analysis, using serum CRP levels or serum BAFF levels, respectively, to separate severe from nonsevere cases results in the best test characteristics (Figure 4B, CRP: AUC 0.948, BAFF: AUC 0.843). Using a CRP level of 271.6 mg/l as the cut-off value results in a sensitivity of 85.7% and specificity of 93.3%. In the case of serum BAFF levels a cut-off value of 1506 pg/ml (values above or equal) to seperate severe from nonsevere cases results in a sensitivity of 85.7% and a specificity of 70.0%. Leucocyte counts (AUC 0.676), serum IL-6 (AUC 0.533), or PCT (AUC 0.836) showed less favorable test characteristics (data not shown).

Taken together, results up to this point support the hypothesis that BAFF has characteristics of an acute phase protein like CRP and shows prognostic value in acute pancreatitis.

## Discussion

So far, BAFF was mainly recognized as a marker in autoimmune diseases, foremost SLE. However, hints in the literature like increased BAFF serum levels in patients with some viral infections and our presented data now suggest that BAFF is important in inflammatory processes in a more general way. Using acute pancreatitis as a model for severe systemic inflammation without confounding factors like infectious agents, autoimmune processes, or malignant processes, we were able to show for the first time that BAFF behaves like an acute phase protein with a kinetic similar to IL-6 or PCT.

Because the course of disease is highly variable it is of great clinical value to have simple predictive parameters measured as early as possible to determine, which patients need special attention and will develop complications. There are some established scoring systems to achieve this goal, like the Ranson score or APACHE II score [9]. However, besides the rather time consuming procedures to calculate these scores using many different parameters, the most significant problem is that they need observation of the patients for up to 48 h before a value can be determined. Parameters that can be determined once at the time-point of admission and allow the prediction of the course of acute pancreatitis would be preferable for clinical guidance. Therefore, many studies tried to identify parameters that can be measured early on and will predict the severity of the course of disease (reviewed in [12]). However, no optimal parameter was found so far. Some studies reported PCT as a potential candidate to predict severe disease within the first 24 h [13], [14]. However, other studies dismissed the predictive value at least for infection of necrosis [15], which in most cases is the basis for a severe course of disease. Our results show less predictive value for PCT measured at the day of admission, as compared to BAFF or CRP, respectively. We did not analyze the predictive value of BAFF or CRP serum concentration measured at later time points, because this would not aid in early management of patients.

Overall CRP seems to be the best characterized predictive parameter in acute pancreatitis and therefore is the established “gold standard” when testing new potential predictive parameters [12]. CRP levels have been reported more useful in monitoring disease activity than predicting the course of disease, mainly due to a 48 h delayed increase in CRP in patients that will develop severe pancreatitis [12]. However, after stratification of patients in EP, NP, or sNPoD we found CRP serum levels elevated in patients that develop severe disease (sNPoD) from the time-point of admission throughout the first nine days. Together with serum BAFF levels, CRP levels at admission were the best predictive parameter for developing sNPoD. Elevated CRP levels at admission might be due to a delayed admission to the hospital after the onset of symptoms. However, most patients included in the analysis had symptoms no longer than 24 h.

An additional value of the assessment of CRP is the distinction between necrotizing and edematous pancreatitis regardless of the severity of disease. Such a discriminatory capacity of CRP was described in some studies before [16], [17] and confirmed in the present study (Figure 3B). Additionally, we show that simple leucocyte counts also have a great value in monitoring disease activity and recognizing patients that will develop complicated necrotizing disease. Although the predictive capacity is low at admission, leucocyte counts will steadily increase in patients with bad prognosis from day 4 after admission, which allows again a distinction between patients with mere necrotizing pancreatitis and patients with complicated necrotizing pancreatitis (sNPoD).

With respect to BAFF serum levels, this is the first study to show a role for BAFF in acute inflammation as an acute phase reactant and its predictive value in acute pancreatitis, which outperforms established parameters like IL-6, PCT, and leucocyte counts.

Additionally, and in contrast to CRP where the exact physiological role in the inflammatory processes is less clear, the role of BAFF in inflammation is better defined (reviewed in [3]). One important function is the extension of the B cell pool. BAFF initiates a survival signal in transitional B cells, leading to an expansion of the B cell pool [3]. This expansion of the B cell pool is important during acute inflammatory processes, because not only the number of B cells, but also the number of detectable antigens increase due to a less stringent selection at the transitional B cell stage. Of course, the downside of the latter is, that more B cells will survive baring BCRs that detect autoantigens. This explains the positive relationship between BAFF levels and severity of some autoimmune diseases, like systemic lupus erythematodes or autoimmune pancreatitis [18]. Taken together with our data, the role of BAFF can be interpreted as “emergency cytokine” or acute phase reactant - the organism tolerates more autoreactive B cells to gain an expanded spectrum of BCRs, increasing the antigen-detecting capacity of the B cell pool in an acute inflammatory response.

It has been shown before, that BAFF serum levels are elevated in patients with autoimmune pancreatitis (AIP), a special case of acute pancreatitis of autoimmune origin with increase of IgG4 [18]. The study compared patients with AIP, chronic pancreatitis, pancreatic cancer and healthy subjects and found elevated levels of serum BAFF in AIP as compared to all other entities. The same study showed that BAFF serum levels correlate with IgG4 levels and after adequate treatment of AIP with steroids, BAFF levels decreased to normal. The authors argue for a special role of BAFF in the pathogenesis of AIP, however a control group of patients with acute pancreatitis of other causes (e.g. alcoholic, biliary, etc. ) was not included. It might still be that there is a special role for BAFF in AIP, which supports disease progress, e.g. by increasing the switch of B cells to IgG4 producing cells, however, our present data suggest that acute pancreatitis regardless of the cause leads to an increase in BAFF serum levels due to an acute phase reaction. To clarify if BAFF plays a specific role in the pathogenesis of AIP it would be necessary to show that BAFF serum levels are elevated in asymptomatic patients before the onset of autoimmune pancreatitis. However, the observation that BAFF serum levels return to normal after successful treatment of AIP with steroids, favors a scenario where the increase in BAFF serum levels is merely an epiphenomenon due to systemic inflammation.

In summary, BAFF behaves like an acute phase reactant and outperforms established markers of inflammation in acute pancreatitis, like IL-6 and PCT underscoring the role of BAFF in the acute inflammatory response. However, it needs to be emphasized that our study also confirmed CRP as the “gold standard” for predicting severity of acute pancreatitis. Never the less, this study supports the view of BAFF as an acute phase reactant and general marker of inflammation and argues against BAFF as mere “autoimmune cytokine”.
